# Difficult weaning in delayed onset diaphragmatic hernia

**DOI:** 10.4103/0974-2700.50750

**Published:** 2009

**Authors:** Syed Moied Ahmed, Abu Nadeem, Jyotishka Pal, Rahul Gupta, Sunil Chauhan

**Affiliations:** Department of Anaesthesiology and Critical Care, JN Medical College, Aligarh Muslim University, Aligarh, India

**Keywords:** Diaphragm, hernia, and difficult weaning

## Abstract

Diaphragmatic injuries are relatively rare and result from either blunt or penetrating trauma. Regardless of the mechanism, diagnosis is often missed and high index of suspicion is vital. The clinical signs associated with a diaphragmatic hernia can range from no outward signs to immediately life-threatening respiratory compromise. Establishing the clinical diagnosis of diaphragmatic injuries (DI) can be challenging as it is often clinically occult. Accurate diagnosis is critical since missed DI may result in grave sequelae due to herniation and strangulation of displaced intra-abdominal organs. We present a case of polytrauma with rib fracture and delayed appearance of diaphragmatic hernia manifesting as difficult weaning from ventilatory support.

## INTRODUCTION

The diaphragm is the major muscle of respiration. Spontaneous breathing relies primarily on diaphragmatic excursion to produce negative intrathoracic pressure. Whenever diaphragmatic function decreases, concomitant respiratory dysfunction ensues. Blunt and penetrating injury to the diaphragm is one of the commonest causes of acquired diaphragmatic hernias. Delay in detecting and repairing diaphragmatic injury increases both morbidity and mortality.

## CASE REPORT

A 46-year-old male presented to the casualty with severe respiratory distress associated with chest pain following an alleged history of fall from height. The patient was provisionally diagnosed as a case of blunt trauma chest with bilateral hemothorax with right-sided fracture clavicle. An urgent X-ray chest was advised. Bilateral ICTD were inserted, which resulted in drainage of approximately 600 ml bloody fluid from each ICTD. With the patient developing hypotension, tachypnoea (> 36 breaths/min) and a deteriorating Glasgow Coma Scale (GCS), the patient was intubated and shifted to ICU.

The patient was sedated and ventilatory support was initiated with volume assist control mode initial settings being tidal volume (V_T_) of 8 ml/kg, respiratory rate(RR) of 14, inspiratory flow rate (IFR) of 60 L/min, positive end expiratory pressure (PEEP) of 8 cm of H_2_O and FiO_2_of 1. X-ray chest confirmed fracture of right clavicle and right-sided 3^rd^, 4^th^ and 5^th^ ribs. Both the costophrenic angles were obliterated with the bilateral ICTD drain in situ. Fluids and vasoactive agents were administered so as to obtain central venous pressure (CVP) of > 12 cm H_2_O and maintain a mean arterial pressure (MAP) of >65 mmHg. In accordance with arterial blood gas (ABG) analysis after a period of 6 h, which showed a PaO_2_ of 200 mmHg, PaCO_2_ of 42 mmHg, pH of 7.37 and HCO_3_ of 21.3 mEq/L, over the next 18 h, the FiO_2_ was gradually reduced to 0.5 and the PEEP was increased to 10 cm H_2_O as the MAP increased to 75 mmHg. Within 48 h of ICU admission, the vasoactive support was gradually tapered off and enteral feeding was initiated. Over the next 48 h (4th ICU day), the requirement of PEEP was reduced to 5 cm H_2_O, minute ventilation (V_E_) was reduced to < 10 l with a maintained PaO_2_/FiO_2_ ratio of > 350. As there was no fresh drainage and the X-ray chest showed fully expanded lungs, both the ICTD were removed. Synchronized intermittent mode (SIMV) was initiated with a back up rate of 12, V_T_ of 450 ml, PS of 10 cm H_2_O and a PEEP of 5 cm H_2_O. On the 6^th^ ICU day, the patient developed ventilator-associated pneumonia (VAP).

Over the next 2 days (9th ICU day), he remained haemodynamically stable but his ventilatory requirement increased (FiO_2_ = 0.6, RR = 14, PS = 12) so as to maintain a PaO_2_/FiO_2_ ratio of > 300. Because of the anticipated prolonged mechanical ventilation, a percutaneous tracheostomy (PCT) was planned. By the 15th day, as the VAP resolved, it was decided to wean the patient from the ventilator. Over the next 24 h (16th day), the PEEP was reduced to 6, PS was reduced to 8 cm H_2_O and the FiO_2_ was maintained at 0.4. The patient was generating a V_T_ of > 400 ml with a PS of 8 cm H_2_O and the spontaneous RR was ranging between 8 and 14/min. With these ventilatory settings, the patient was maintaining a rapid shallow breathing index (RSBI) of < 100. The patient was then switched over to CPAP/PSV (PS of 8 cm H_2_O, PEEP of 4 cm H_2_O) on the 17th day and was allowed to remain in the same settings for the next 24 h during which no signs of exhaustion or distress were manifested. His RR ranged between 15 and 19/min, generating a V_T_ of > 450 ml. Considering the past 24-h ventilatory record and the morning ABG, the patient was detached from the ventilator and O_2_ was administered @ 3L/min through Ayres T piece connected to the tracheostomy tube. Within 30 min of detachment from the ventilatory support, the patient became exhausted, dyspneic with RR of > 28/min and a SpO_2_ of < 92%. Considering it to be respiratory failure due to muscle weakness from prolonged ventilatory support and nutritional deficiency, the patient was reverted back to the ventilatory support with the previous settings. It was decided to give repeated trials after every 4 h with a PS of 6 cm H_2_O and gradually increase the duration of trial from 30 min to the maximum time tolerated. The patient remained comfortable with the ventilatory support but deteriorated when detached from the ventilator. Despite 24 h of exercise with that protocol, the ventilatory support could not be reduced. A thorough clinical examination and investigation was done to rule out any lung pathology. On auscultation, air entry was decreased over the left lower chest. X-ray revealed opaque shadow along with the appearance of the distal end of the nasogastric tube over the left lower chest. The presence of the nasogastric tube was confirmed with the presence of gurgling sound on auscultation after administration of air through the nasogastric tube. CTVS Surgeon was referred who diagnosed the case as delayed onset diaphragmatic hernia following blunt trauma chest. The patient was planned for hernia repair by a left thoracotomy approach. Peroperative findings were left pleural space in lower part filled with distended stomach (fundus and part of body) and part of spleen. A single rent of size 30 cm^2^ was present in the left dome of diaphragm, which was repaired.

Postoperatively, the patient was electively ventilated for 24 h and gradually weaned and finally detached from the ventilator after 48 h under the cover of O_2_ supplementation through Ayre's T piece @ 3 L/min. The patient fared well after removal from the ventilatory support with occasional O_2_ supplementation. The patient was decannulated after performing cuff leak test of the tracheostomy tube on the 26th ICU day. He was observed in the ICU for the next 24 h and was shifted to the surgical floor without any further complication.

## DISCUSSION

Diaphragmatic injuries are relatively rare and result from either blunt or penetrating trauma. These injuries were described first by Sennertus in 1541. Riolfi performed the first successful repair in 1886. Not until 1951, when Carter *et al.*, published the first case series, was this injury well understood and delineated.[1] Diaphragmatic hernias can be divided into two categories: congenital defects and acquired defects.

Approximately 0.8%–1.6% of patients with blunt trauma show a rupture in the diaphragm.[2] The male-to-female ratio is 4:1, with most presenting in the third decade of life. Blunt trauma accounts for 75% of ruptures, and penetrating trauma accounts for the rest. Approximately 69% of hernias are left-sided, 24% are right-sided, and 15% are bilateral.[3,4] Children have equal rates of rupture per side, likely due to laxity of liver attachments.[5]

Patients with delayed diaphragmatic herniation frequently present months to years after the initial injury with manifestations of visceral incarceration, obstruction, ischemia from strangulation, or perforation. In our case, it was the difficulty in weaning off mechanical ventilatory support that subsequently led to a suspicion and diagnosis of diaphragmatic herniation.

Symbas *et al.*, observed a delay in diagnosis in 8% of cases of diaphragmatic injury from 18 h to 15 years after injury.[6] Chest radiography is the standard in the advanced trauma life support (ATLS) protocol for a trauma workup. Approximately 23–73% of traumatic diaphragmatic ruptures will be detected by initial chest radiograph, with an additional 25% found with subsequent films. Chest radiograph is most sensitive investigation for detecting left-sided hernias. Chest radiographic findings that indicate traumatic rupture include the following:[5]

1. Abdominal contents in the thorax, with or without signs of focal constriction (“collar sign”)
2. Nasogastric tube seen in the thorax,
3. Elevated hemidiaphragm (>4 cm higher on left vs right) and
4. Distortion of diaphragmatic margin.

Conventional CT scan has been reported to have a sensitivity of 14%–82%, with a specificity of 87%. Helical CT increased sensitivity 71-100%, with higher sensitivity for left vs right. CT findings indicating rupture include the following:

1. Direct visualization of injury
2. Segmental diaphragm non-visualization
3. Intrathoracic herniation of viscera
4. Collar sign”
5. Peridiaphragmatic active contrast extravasation.[5]

Ultrasonography (focused assessment with sonography for trauma [FAST] scan) has been reported to detect diaphragmatic hernias.[7] During the visualization of each upper quadrant, the movement of the diaphragm was noted to be decreased in patients with diaphragmatic hernias. This technique is limited in patients who are on mechanical ventilation because of the positive pressure of the thoracic cavity.[7]

Surgery is mainstay in the treatment of diaphragmatic rupture.[8] Operative repair is technically more difficult if the surgery is delayed. The difference is based on the degree of adhesion present in the thoracic cavity and the state of the herniated organs. After reduction of the abdominal contents, the diaphragm can usually be repaired simply with monofilament nonabsorbable suture.

The pressure difference between the abdominal peritoneum and pleural cavities is 7–20 cm of water in quite breathing but could be as high as 100 cm of water in forced respiration.[1] When diaphragm ruptures, this pressure difference causes the abdominal viscera to herniate through the defect into the chest cavity.[9]

In this case, while the patient was on mechanical ventilation, the intrathoracic pressure was constantly higher than the intra abdominal pressure due to the effect of positive pressure ventilation. However, as long as the patient was on CPAP/PSV, the patient did not manifest any symptoms of respiratory embarrassment. It was only when the patient was detached from the ventilatory support that the effects of spontaneous respiratory efforts created the negative intrathoracic pressure that led to the herniation of the intra abdominal contents through the occult diaphragmatic defect in accordance to the pressure gradient between the abdominal and thoracic cavities.

## CONCLUSION

Diaphragmatic hernia can often be missed. A correct diagnosis remains difficult and is usually made late. Accurate diagnosis is critical however as a missed DI may result in grave sequelae due to herniation and strangulation of displaced intra-abdominal organs. Blunt diaphragmatic rupture (DR) is a rare condition usually masked by multiple associated injuries, which are the main cause of morbidity and mortality. Physicians should consider the diagnosis in patients who have a history of blunt trauma to the chest or abdomen and develop gastrointestinal or respiratory symptoms. The chest x-ray, especially when performed after insertion of a nasogastric tube, is a useful screening tool.[10] Single or serial plain chest radiographs with a high index of suspicion are diagnostic in most cases. Respiratory distress should be treated with intubation as intercostal drainage (ICD) may not improve the situation and is associated with a high risk of iatrogenic injuries. Even difficulty in weaning off ventilatory support can serve as a clue to the underlying occult defect. Surgical repair is mandatory, and laparotomy should be the preferred approach in unstable patients. To avoid missed injury thorough inspection of both hemidiaphragms should be done routinely on every trauma patient undergoing laparotomy.
